# A DR-*ℓ*_1_-PRLS Approach to Adaptive Equalization in Single-Carrier UWA Communication

**DOI:** 10.3390/s26092775

**Published:** 2026-04-29

**Authors:** Xiao-Chen Chen, Guan-Quan Dai, Yang Shi, Fei-Yun Wu

**Affiliations:** 1 College of Navigation, Jimei University, Xiamen 361021, China; 202411823026@jmu.edu.cn (X.-C.C.); 202514823003@jmu.edu.cn (G.-Q.D.); 202512861046@jmu.edu.cn (Y.S.); 2 College of Transportation and Navigation, Quanzhou Normal University, Quanzhou 362000, China

**Keywords:** underwater acoustic communication, sparse adaptive equalization, data reuse, proportionate recursive least squares, ℓ1 regularization

## Abstract

In single-carrier underwater acoustic (UWA) communication systems, sparse multipath channels and long delay spreads pose significant challenges to adaptive equalization, often leading to limited steady-state accuracy and degraded detection performance. To address this issue, this paper proposes a data reuse-based $\ell_{1}$-regularized proportionate recursive least-squares algorithm (DR-$\ell_{1}$-PRLS) for sparse adaptive equalization. The proposed method incorporates a data reuse (DR) mechanism into the $\ell_{1}$-PRLS framework, enabling multiple equivalent uses of each received–reference sample pair without increasing pilot overhead. Meanwhile, by combining the proportionate update strategy with the $\ell_{1}$ sparsity constraint, the structural information of sparse channels can be more fully exploited, thereby improving parameter estimation accuracy. Numerical simulations are conducted to evaluate the proposed method in terms of convergence behavior, tracking capability, computational complexity, and bit error rate (BER), and comparisons are made with LMS, RLS, PRLS, $\ell_{1}$-PRLS, and DR-PRLS algorithms. Simulation results show that, under sparse underwater acoustic channel conditions, DR-$\ell_{1}$-PRLS achieves lower steady-state error and better BER performance while maintaining good tracking capability, thereby demonstrating its effectiveness and robustness for sparse adaptive equalization in single-carrier underwater acoustic communications.

## 1. Introduction

Single-carrier transmission remains attractive for underwater acoustic communications because it can be combined with relatively simple receiver structures and effective equalization [1] strategies. However, in practical underwater acoustic (UWA) channels, long delay spread, severe multipath, and Doppler effects [2] make reliable transmission particularly difficult. Therefore, robust equalization [3] remains a key requirement for single-carrier UWA systems [4].

UWA channels [5] are also typically characterized by sparse multipath propagation, where only a few dominant paths (or several clusters) [6] contain most of the channel energy. In such environments, adaptive equalization is essential for mitigating severe inter-symbol interference (ISI) under limited training and time-varying conditions. Moreover, when the underlying system (or the equivalent equalizer) exhibits sparse or cluster-sparse structures [7], explicitly exploiting sparsity priors [8,9] in the adaptive update can substantially improve convergence behavior and steady-state accuracy, especially with short training sequences or under low-signal-to-noise-ratio conditions.

Consequently, sparse-aware adaptive algorithms [10] have attracted considerable attention for UWA communications. Existing approaches include LMS-type methods [11,12] with sparsity-promoting penalties (e.g., $\ell_{0}/\ell_{1}$-type regularization) and proportionate updates such as PNLMS/IPNLMS [13,14], which can improve convergence and tracking in sparse systems. Nevertheless, LMS-based algorithms are often limited by step size selection [15], making it difficult to simultaneously achieve fast convergence and low steady-state misadjustment in highly dynamic UWA scenarios.

In contrast, recursive least-squares (RLS) algorithms [16] exploit second-order statistical information and typically provide faster convergence and stronger tracking capability, which are attractive for rapidly time-varying UWA channels [17]. To further leverage sparsity, the proportionate update mechanism [18] was introduced into the RLS framework, leading to the proportionate recursive least-squares (PRLS) algorithm [19]. Its performance advantages over conventional RLS have been analyzed from both transient and steady-state perspectives, establishing a foundation for sparse RLS design. Building on PRLS, sparsity-regularized variants [20] such as $\ell_{1}$-regularized PRLS (and related approximations) [21] strengthen the exploitation of sparse (or nearly sparse) structures and can further suppress steady-state error.

Although $\ell_{1}$-PRLS can combine the fast convergence of RLS with sparsity-aware adaptation, its steady-state residual error, re-convergence capability, and tracking performance may still be limited under low-SNR conditions, short training sequences, and rapidly time-varying UWA channels [22,23]. In contrast, DR-based proportionate methods such as DR-PRLS can improve observation utilization and often accelerate error reduction, though, without explicit sparse regularization, their steady-state estimation accuracy in highly sparse or cluster-sparse channels may remain unsatisfactory [24,25]. Therefore, existing $\ell_{1}$-PRLS and DR-PRLS schemes improve different aspects of sparse adaptive equalization, though neither can simultaneously provide fast convergence, low steady-state error, and robust tracking performance in challenging UWA environments [26].

This highlights the need for a unified adaptive framework that can jointly exploit sparse priors and improve observation utilization for sparse time-varying UWA channels.

Motivated by this gap, this paper proposes a data reuse-based $\ell_{1}$-regularized proportionate recursive least-squares (DR-$\ell_{1}$-PRLS) algorithm for sparse adaptive equalization [27] in single-carrier UWA communications, aiming to improve equalization accuracy and robustness in sparse/cluster-sparse UWA channels [28].

More importantly, the performance gain of the proposed method is not a simple stacking of DR and $\ell_{1}$ regularization. Compared with DRPRLS, which mainly reduces the residual error by reusing the same snapshot, DR-$\ell_{1}$-PRLS incorporates sparsity attraction into the DR-equivalent recursion and yields a closed-form single-step update. In this equivalent form, DR reshapes the effective proportionate RLS correction through a scalar gain factor (denoted as $\beta_{p}\left(n\right)$), which strengthens per-snapshot error cancellation. When $|\alpha_{p}\left(n\right)|<1$, the inner-loop residual decreases geometrically with the reuse factor $N_{r}$, thereby lowering the misadjustment (error floor) without increasing pilot overhead. In contrast to conventional $\ell_{1}$-PRLS, where steady-state improvement relies primarily on coefficient shrinkage, the proposed DR-$\ell_{1}$-PRLS further improves steady-state accuracy via DR-induced gain shaping while preserving a compact RLS-style recursion suitable for real-time UWA receivers. Therefore, the additional gain over existing DR-PRLS and regularized RLS variants stems [29] from the interaction between DR-induced gain shaping and $\ell_{1}$-based sparse attraction within an equivalent single-step recursion, rather than from a purely combinational modification.

The main contributions of this paper are summarized as follows:

- A DR-$\ell_{1}$-PRLS algorithm for sparse adaptive equalization is proposed. The DR mechanism is incorporated into the $\ell_{1}$-PRLS framework so that each group of received observations can be equivalently reused within the same update period. By jointly exploiting $\ell_{1}$-based sparsity constraints and proportionate updates, the proposed method improves coefficient estimation accuracy and steady-state performance.
- The equivalent update derivation and implementation under the DR mechanism are provided. A closed-form equivalent single-step update expression is derived, and practical guidelines for selecting the reuse factor $N_{r}$ are given. The effects of DR on convergence behavior, steady-state error, and tracking capability are also discussed.
- Computational complexity analysis and simulation validation are conducted. The per-iteration computational complexity is analyzed, and simulations compare the proposed method with LMS, RLS, PRLS, DR-PRLS, and $\ell_{1}$-PRLS in terms of mean square deviation (MSD), tracking performance, and bit error rate (BER), thereby verifying its effectiveness and robustness in sparse UWA channels.

The remainder of this paper is organized as follows: Section 2 presents the single-carrier UWA system model and problem formulation. Section 3 introduces the proposed DR-$\ell_{1}$-PRLS algorithm, including parameter settings and complexity analysis. Section 4 provides simulation results and discussions. Section 5 concludes this paper.

## 2. Single-Carrier UWA System Model and Sparse Adaptive Equalization Problem Formulation

This section follows the receiver processing flow of single-carrier underwater acoustic (UWA) communications. First, a discrete-time complex baseband system model is established. Then, the sparse adaptive equalization problem is formulated, which provides the foundation for the DR-$\ell_{1}$-PRLS algorithm proposed in Section 3.

### 2.1. Single-Carrier UWA Communication System Model

We consider a discrete-time complex baseband single-input single-output (SISO) single-carrier UWA communication system. Let x(n) denote the transmitted symbol at time instant *n*. After propagating through a time-varying UWA channel with $L_{h}$ effective multipath components, the received complex baseband signal can be expressed as

(1) $$y\left(n\right)=e^{j\phi (n)}\sum_{l=0}^{L_{h}-1}h\left(n,l\right)\;x\left(n-l\right)+v\left(n\right)$$

where h(n,l) denotes the equivalent complex channel coefficient of the *l*th path at time *n*, ϕ(n) represents the common phase fluctuation caused by carrier phase drift and residual Doppler effects, and v(n) is the additive noise term (typically modeled as zero-mean complex Gaussian white noise).

According to the pilot-assisted equalization procedure at the receiver, each received frame can be divided into a training segment and a data segment. The training segment is used to adaptively update the equalizer parameters, and the updated equalizer coefficients are then applied to equalize and detect the data segment. To this end, the tapped observation vector at time *n* is constructed as

(2) $$r\left(n\right)={\left[y\left(n+K_{1}\right),\ldots ,y\left(n-K_{2}\right)\right]}^{T}$$

where $K_{1}$ and $K_{2}$ denote the numbers of forward and backward taps, respectively, and $r\left(n\right)\in C^{(K_{1}+K_{2}+1)\times 1}$.

For a linear equalizer (LE), the output is given by

(3) $$\hat{x}\left(n\right)=e^{-j\hat{\phi }\left(n\right)}w_{f}^{H}\left(n\right)r\left(n\right)$$

where $w_{f}\left(n\right)$ is the feedforward equalizer coefficient vector and $\hat{\phi }\left(n\right)$ is the phase estimate.

When a decision feedback equalizer (DFE) is employed, a feedback branch is further introduced to suppress post-cursor inter-symbol interference (ISI), and the output becomes

(4) $$\hat{x}\left(n\right)=e^{-j\hat{\phi }\left(n\right)}\left(w_{f}^{H}\left(n\right)r\left(n\right)+f^{H}\left(n\right){\hat{x}}_{b}\left(n\right)\right)$$

where $f\left(n\right)\in C^{K_{3}\times 1}$ is the feedback filter coefficient vector, $K_{3}$ is the number of feedback taps, and

(5) $${\hat{x}}_{b}\left(n\right)={\left[\hat{x}\left(n-1\right),\ldots ,\hat{x}\left(n-K_{3}\right)\right]}^{T}.$$

To maintain consistency with the subsequent adaptive algorithm derivation, both LE and DFE are represented in a unified “regressor vector–coefficient vector” form by defining

(6) $$u\left(n\right)=\left\{\begin{array}{cc} r(n), & \mathrm{LE}\\ \left[\begin{array}{c} r(n)\\ {\hat{x}}_{b}\left(n\right) \end{array}\right], & \mathrm{DFE} \end{array}\right.\;w\left(n\right)=\left\{\begin{array}{cc} w_{f}\left(n\right), & \mathrm{LE}\\ \left[\begin{array}{c} w_{f}\left(n\right)\\ f(n) \end{array}\right], & \mathrm{DFE} \end{array}\right.$$

so that the equalizer output can be written in a unified linear estimation structure in the adaptive update stage.

**Figure 1:**
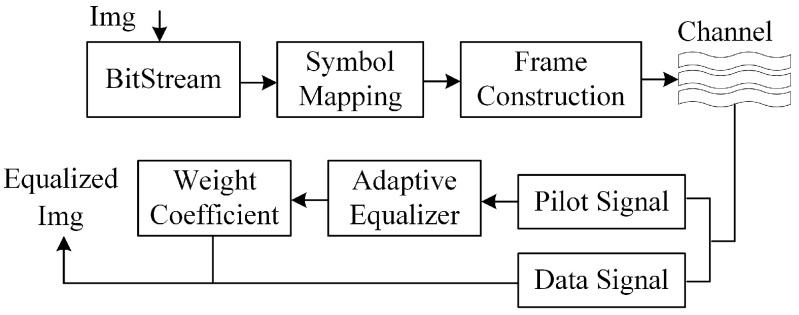
Complex baseband receiver structure of the single-carrier UWA communication system.

For clarity, Figure 1 shows the complex baseband receiver structure of the single-carrier UWA communication system. After modulation, the transmitted symbols pass through a time-varying sparse multipath UWA channel. At the receiver, complex baseband sampling, phase compensation, observation vector construction, and symbol recovery are performed by using either a linear equalizer (LE) or a decision feedback equalizer (DFE).

In the pilot-assisted mode, the training segment is used to adaptively update the equalizer parameters. The updated coefficients are then applied to detect the data segment. This block diagram is consistent with the signal model in (1)–(6) and provides an intuitive reference for the sparse adaptive equalization formulation in the following subsection.

### 2.2. Sparse Adaptive Equalization Problem Formulation

In adaptive equalization, the desired signal at the receiver can be modeled as

(7) $$d\left(n\right)=w_{o}^{H}u\left(n\right)+v\left(n\right)$$

where $w_{o}\in C^{L_{e}\times 1}$ denotes the optimal (or equivalent) equalizer coefficient vector to be estimated, $u\left(n\right)\in C^{L_{e}\times 1}$ is the unified input regressor vector formed from the received samples (and feedback decisions in the DFE case), v(n) is the observation noise term, and $L_{e}$ is the equalizer parameter dimension.

Since UWA channels usually exhibit sparse or cluster-sparse structures, most of the multipath energy is concentrated on a few dominant paths or several path clusters. As a result, the equivalent equalizer coefficients are often approximately sparse as well. If sparse prior constraints are explicitly incorporated into the adaptive update, lower steady-state error and better estimation accuracy can be achieved under limited-training-length, low-SNR, or fast time-varying conditions.

Define the a priori error as

(8) $$e\left(n|n-1\right)=d\left(n\right)-w^{H}\left(n-1\right)u\left(n\right)$$

where w(n−1) is the equalizer coefficient estimate at the previous time instant. The method proposed in this paper is developed under this error-driven framework, combining proportionate updating, second-order RLS statistics, and sparse regularization to improve adaptive equalization performance over sparse UWA channels.

## 3. Proposed DR-$\ell_{1}$-PRLS Algorithm

Based on the unified sparse adaptive equalization framework established in Section 2, this section develops the proposed DR-$\ell_{1}$-PRLS algorithm. For brevity, the signal model and the definition of the a priori error are omitted, and we use the same notation as in Section 2 throughout; namely, the regressor is denoted by u(n), the desired signal by d(n), and the a priori error by e(n∣n−1). The derivation starts from the conventional $\ell_{1}$-PRLS recursion and then introduces a data reuse (DR) mechanism to improve steady-state estimation accuracy under noisy UWA conditions without increasing pilot overhead.

### 3.1. Baseline $\ell_{1}$-PRLS Recursion and DR Motivation

Let $w\left(n\right)\in C^{L_{e}\times 1}$ denote the equalizer coefficient vector at time index *n*, where $L_{e}$ is the equalizer length. Let $u\left(n\right)\in C^{L_{e}\times 1}$ be the input vector, and let d(n)∈C be the desired signal. The a priori error is

(9) $$e\left(n|n-1\right)=d\left(n\right)-w^{H}\left(n-1\right)u\left(n\right).$$

The $\ell_{1}$-PRLS update is

(10) $$w\left(n\right)=w\left(n-1\right)+G\left(n-1\right)k\left(n\right)e^{*}\left(n|n-1\right)-\xi \;P\left(n\right)\;{\mathrm{sgn}}_{\epsilon }\;\left(w(n-1)\right).$$

where ξ>0 is the sparsity regularization factor, $P\left(n\right)\in C^{L_{e}\times L_{e}}$ is the inverse correlation matrix, $k\left(n\right)\in C^{L_{e}\times 1}$ is the RLS gain vector, and $G\left(n-1\right)\in R^{L_{e}\times L_{e}}$ is the diagonal proportionate matrix.

The RLS gain and inverse correlation matrix are updated as

(11) $$k\left(n\right)=\frac{P(n-1)u(n)}{\lambda +u^{H}\left(n\right)P\left(n-1\right)u\left(n\right)},$$

(12) $$P\left(n\right)=\lambda^{-1}\;\left[P\left(n-1\right)-k\left(n\right)u^{H}\left(n\right)P\left(n-1\right)\right],$$

where λ∈(0,1] is the forgetting factor.

In this work, the proportionate rule follows the conventional form commonly used in PRLS/$\ell_{1}$-PRLS-type sparse adaptive filters. The specific parameter values used in the experiments are listed in Table 1, while their roles are stated here for clarity. The *k*th diagonal entry of G(n−1) is defined as

(13) $$g_{k}\left(n-1\right)=\frac{\mu (1-\alpha )}{2L_{e}}+\mu \left(1+\alpha \right)\frac{|w_{k}\left(n-1\right)|}{{2∥w\left(n-1\right)∥}_{1}+\varepsilon_{g}},\;k=1,2,\ldots ,L_{e}.$$

where μ>0 is a scaling factor, α∈[−1,1] controls the balance between uniform and proportionate adaptation, ${∥w\left(n-1\right)∥}_{1}=\sum_{i=1}^{L_{e}}\left|w_{i}\left(n-1\right)\right|$ is the $\ell_{1}$ norm of w(n−1), and $\varepsilon_{g}>0$ is a small constant to avoid division by zero. Accordingly, the diagonal proportionate matrix is given by

(14) $$G\left(n-1\right)=\mathrm{diag}\;\left\{g_{1}\left(n-1\right),g_{2}\left(n-1\right),\ldots ,g_{L_{e}}\left(n-1\right)\right\}.$$

For complex coefficients, the smoothed sign function is defined element-wise as

(15) $${\left[{\mathrm{sgn}}_{\epsilon }\left(w\right)\right]}_{i}=\frac{w_{i}}{|w_{i}|+\epsilon },\;i=1,2,\ldots ,L_{e},$$

where ϵ>0 is a small constant for numerical stability.

This update combines three mechanisms: The RLS recursion exploits second-order information. The proportionate matrix G(n−1) assigns larger updates to large-magnitude taps. The $\ell_{1}$ attraction term promotes sparsity.

However, in noisy and fast time-varying UWA channels, a single update at each time index may not use the current data sufficiently. The main idea of DR is to reuse the same observation pair {u(n),d(n)} several times within the same time index *n*. This can reduce the instantaneous error more fully and improve data utilization without increasing the training length.

**Table 1:** Parameter settings of the considered algorithms.

Alg.	μ	λ	δ	α	$\varepsilon_{G}$	ξ	$N_{r}$
LMS	0.01						
RLS		0.995	100				
PRLS	64	0.995	100	−0.3	$10^{-4}$		
$\ell_{1}$-PRLS	64	0.995	100	−0.3	$10^{-4}$	$10^{-2}$	
DR-PRLS	64	0.995	100	−0.3	$10^{-4}$		3
DR-$\ell_{1}$-PRLS	64	0.995	100	−0.3	$10^{-4}$	$10^{-2}$	3

### 3.2. DR-Based Derivation and Equivalent Single-Step Update

A. Inner-loop DR recursion

Let $N_{r}\in N$ denote the data reuse factor. At time index *n*, the same observation pair {u(n),d(n)} is reused $N_{r}$ times. Define the inner-loop iterates $w^{\left(i\right)}\left(n\right)$, $i=0,1,\ldots ,N_{r}$, with

(16) $$w^{\left(0\right)}\left(n\right)=w\left(n-1\right).$$

For $i=1,2,\ldots ,N_{r}$, define the inner-loop error as

(17) $$e^{\left(i\right)}\left(n\right)=d\left(n\right)-{\left(w^{\left(i-1\right)}\left(n\right)\right)}^{H}u\left(n\right),$$

and update

(18) $$w^{\left(i\right)}\left(n\right)=w^{\left(i-1\right)}\left(n\right)+G\left(n-1\right)k\left(n\right){\left(e^{\left(i\right)}\left(n\right)\right)}^{*}-\xi \;P\left(n\right)\;{\mathrm{sgn}}_{\epsilon }\;\left(w^{\left(i-1\right)}\left(n\right)\right).$$

The final output is

(19) $$w\left(n\right)=w^{\left(N_{r}\right)}\left(n\right).$$

A direct implementation requires explicit inner iterations. To reduce complexity, an equivalent single-step form is derived below.

B. Freezing approximation

To obtain a compact recursion, we adopt a within-snapshot freezing approximation. Since all inner updates use the same sample pair, and since $N_{r}$ is usually small in practice, the within-snapshot variation of the coefficient vector is limited. As a result, the variation of the proportionate matrix is also small within the inner loop. Therefore, G(n−1) is treated as fixed, and the sparse attraction term is approximated by

(20) $${\mathrm{sgn}}_{\epsilon }\;\left(w^{\left(i-1\right)}\left(n\right)\right)\approx {\mathrm{sgn}}_{\epsilon }\;\left(w(n-1)\right).$$

The freezing approximation is accurate when the coefficient variation inside one snapshot is small. This usually holds when the reuse factor $N_{r}$ is small and each inner update causes only a limited change in the coefficient vector. It is also more reasonable when the regularization parameter ξ is not large, so that the sparse attraction term does not vary strongly within the inner loop. Under these conditions, both G(n−1) and ${\mathrm{sgn}}_{\epsilon }\;\left(w^{\left(i-1\right)}\left(n\right)\right)$ remain nearly unchanged during the $N_{r}$ reuse steps.

The main purpose of this approximation is to simplify the multi-step inner recursion into a compact single-step form. This greatly reduces the implementation cost. The induced approximation error remains small when the within-snapshot variation is limited. In contrast, if $N_{r}$ is large, ξ is strong, or the estimation error changes sharply, the mismatch may become more visible. In such cases, the approximation may slightly affect the transient behavior and the final steady-state accuracy. For the parameter settings considered in this work, this effect is found to be small and does not change the main performance trends.

Define

(21) q(n)≜G(n−1)k(n),

(22) $$z\left(n\right)≜\xi \;P\left(n\right)\;{\mathrm{sgn}}_{\epsilon }\;\left(w(n-1)\right),$$

where q(n) is the effective proportionate gain vector and z(n) is the sparse attraction term. Then, the inner update becomes

(23) $$w^{\left(i\right)}\left(n\right)=w^{\left(i-1\right)}\left(n\right)+q\left(n\right){\left(e^{\left(i\right)}\left(n\right)\right)}^{*}-z\left(n\right).$$

C. Inner-loop error recursion

Using the compact update, the inner-loop error satisfies

(24) $${\left(e^{\left(i+1\right)}\left(n\right)\right)}^{*}=\alpha_{p}\left(n\right){\left(e^{\left(i\right)}\left(n\right)\right)}^{*}+b\left(n\right),\;{\left(e^{\left(1\right)}\left(n\right)\right)}^{*}=e^{*}\left(n|n-1\right),$$

where

(25) $$\alpha_{p}\left(n\right)≜1-u^{H}\left(n\right)G\left(n-1\right)k\left(n\right)$$

is the DR contraction factor and

(26) $$b\left(n\right)≜\xi \;u^{H}\left(n\right)P\left(n\right)\;{\mathrm{sgn}}_{\epsilon }\;\left(w(n-1)\right)$$

is the bias term induced by sparse attraction.

For $\alpha_{p}\left(n\right)\neq 1$, the closed-form solution is

(27) $${\left(e^{\left(i\right)}\left(n\right)\right)}^{*}=\alpha_{p}^{\;i-1}\left(n\right)e^{*}\left(n|n-1\right)+\frac{1-\alpha_{p}^{\;i-1}\left(n\right)}{1-\alpha_{p}\left(n\right)}\;b\left(n\right).$$

D. Equivalent single-step update

Summing the inner updates over $i=1,\ldots ,N_{r}$ gives

(28) $$w\left(n\right)=w\left(n-1\right)+q\left(n\right)\sum_{i=1}^{N_{r}}{\left(e^{\left(i\right)}\left(n\right)\right)}^{*}-N_{r}\;z\left(n\right).$$

Define the scalar DR gain factor

(29) $$\beta_{p}\left(n\right)≜\sum_{i=1}^{N_{r}}\alpha_{p}^{\;i-1}\left(n\right)=\left\{\begin{array}{cc} \frac{1-\alpha_{p}^{N_{r}}\left(n\right)}{1-\alpha_{p}\left(n\right)}, & \alpha_{p}\left(n\right)\neq 1,\\ N_{r}, & \alpha_{p}\left(n\right)=1. \end{array}\right.$$

Then,

(30) $$\sum_{i=1}^{N_{r}}{\left(e^{\left(i\right)}\left(n\right)\right)}^{*}=\beta_{p}\left(n\right)e^{*}\left(n|n-1\right)+\frac{N_{r}-\beta_{p}\left(n\right)}{1-\alpha_{p}\left(n\right)}\;b\left(n\right).$$

Substituting the above expressions yields

(31) $$\begin{array}{cc} w(n)\; & \;=w\left(n-1\right)+\beta_{p}\left(n\right)G\left(n-1\right)k\left(n\right)e^{*}\left(n|n-1\right)\\ \; & \;+\frac{N_{r}-\beta_{p}\left(n\right)}{1-\alpha_{p}\left(n\right)}\;G\left(n-1\right)k\left(n\right)b\left(n\right)-N_{r}\xi \;P\left(n\right)\;{\mathrm{sgn}}_{\epsilon }\;\left(w(n-1)\right). \end{array}$$

This is the exact single-step DR-equivalent form under the freezing approximation. It avoids explicit inner iterations. It also shows that DR changes the effective correction gain through the scalar factor $\beta_{p}\left(n\right)$.

### 3.3. Practical Recursion, Implementation, and Discussion

A. Practical recursion

The following practical form is obtained by neglecting the coupling compensation term in the exact single-step update. In practical UWA equalization, the transient behavior is mainly dominated by the proportionate RLS correction term, whereas the $\ell_{1}$ regularization term mainly affects sparsity promotion and steady-state shrinkage. For a compact implementation, the coupling term is therefore omitted.

Let the neglected coupling term be

(32) $$\Delta w_{c}\left(n\right)≜\frac{N_{r}-\beta_{p}\left(n\right)}{1-\alpha_{p}\left(n\right)}\;G\left(n-1\right)k\left(n\right)b\left(n\right),$$

and the dominant error-driven term be

(33) $$\Delta w_{e}\left(n\right)≜\beta_{p}\left(n\right)\;G\left(n-1\right)k\left(n\right)e^{*}\left(n|n-1\right).$$

Their relative magnitude can be bounded by

(34) $$\rho \left(n\right)≜\frac{∥\Delta w_{c}\left(n\right)∥}{∥\Delta w_{e}\left(n\right)∥}\leq \frac{|N_{r}-\beta_{p}\left(n\right)|}{|\beta_{p}\left(n\right)|\;|1-\alpha_{p}\left(n\right)|}\cdot \frac{\left|b(n)\right|}{\left|e(n|n-1)\right|}.$$

The above bound gives a quantitative condition for neglecting the coupling term. Since b(n) is proportional to ξ, the relative magnitude of the coupling term decreases as ξ becomes small. This condition is more likely to hold under moderate-to-high SNR, where |e(n∣n−1)| is less likely to be dominated by rapid noise fluctuations. In addition, when $|1-\alpha_{p}\left(n\right)|$ becomes small, the threshold safeguard forces $\beta_{p}\left(n\right)=N_{r}$, and thus $\Delta w_{c}\left(n\right)=0$.

Therefore, the simplified recursion is mainly suitable for a small ξ, small $N_{r}$, and moderate-to-high SNR. Under the parameter settings used in this work, the above bound indicates that the coupling term remains much smaller than the dominant error-driven correction in most cases. Hence, neglecting this term is unlikely to cause noticeable MSD or BER degradation, but it does lead to a more compact implementation.

The resulting practical DR-$\ell_{1}$-PRLS recursion is

(35) $$w\left(n\right)=w\left(n-1\right)+\beta_{p}\left(n\right)G\left(n-1\right)k\left(n\right)e^{*}\left(n|n-1\right)-N_{r}\xi \;P\left(n\right)\;{\mathrm{sgn}}_{\epsilon }\;\left(w(n-1)\right).$$

B. Why DR improves convergence accuracy

The error recursion in (24) directly explains the role of data reuse. After $N_{r}$ reuses, the inner-loop residual satisfies

(36) $${\left(e^{\left(N_{r}+1\right)}\left(n\right)\right)}^{*}=\alpha_{p}^{N_{r}}\left(n\right)e^{*}\left(n|n-1\right)+\frac{1-\alpha_{p}^{N_{r}}\left(n\right)}{1-\alpha_{p}\left(n\right)}\;b\left(n\right).$$

When the sparse bias term b(n) is relatively small (or effectively compensated by proper selection of ξ), (36) is approximately dominated by the first term, i.e.,

(37) $$\left|e^{\left(N_{r}+1\right)}\left(n\right)\right|\approx |\alpha_{p}{\left(n\right)|}^{N_{r}}\;\left|e\left(n|n-1\right)\right|.$$

Hence, if $|\alpha_{p}\left(n\right)|<1$, DR drives the instantaneous residual down geometrically with respect to $N_{r}$. In other words, the same observation pair contributes a stronger error cancellation effect at each time index, which improves steady-state estimation accuracy (lower misadjustment/error floor) without requiring additional pilot samples.

From the equivalent single-step perspective in (35), DR modifies the effective correction term from

(38) $$\Delta w_{\ell_{1}\text{-}\mathrm{PRLS}}\left(n\right)=G\left(n-1\right)k\left(n\right)e^{*}\left(n|n-1\right)$$

to

(39) $$\Delta w_{\mathrm{DR}}\left(n\right)=\beta_{p}\left(n\right)\;G\left(n-1\right)k\left(n\right)e^{*}\left(n|n-1\right),$$

where the scalar $\beta_{p}\left(n\right)$ is adaptively determined by the geometric series in (29). This mechanism can be interpreted as an adaptive gain shaping the proportionate RLS correction, which enhances per-snapshot error reduction while preserving a compact update form suitable for real-time UWA receivers.

C. Algorithm summary

For clarity, the main procedure of the proposed DR-$\ell_{1}$-PRLS algorithm is briefly described before the detailed implementation is given. At each time instant, the algorithm first evaluates the instantaneous estimation error and then updates the RLS gain and the inverse correlation matrix using the newly received data.

After that, a proportionate matrix is constructed according to the current coefficient magnitudes to assign adaptive update weights. The DR mechanism is then incorporated to improve the utilization efficiency of the current observation, and the coefficient vector is finally updated with an $\ell_{1}$ regularization term to enhance sparsity. The practical implementation is summarized in Algorithm 1.

As shown in Algorithm 1, the proposed method consists of four main operations in each recursion: error computation, recursive information updating, proportionate weighting, and sparsity enhancement.
**Algorithm 1** DR-$\ell_{1}$-PRLS1:**Input:** Desired signal d(n), input vector u(n), forgetting factor λ, regularization parameter δ, filter length $L_{e}$, threshold $\epsilon_{\beta }$, parameter $N_{r}$2:**Output:** Updated coefficient vector w(n)3:Initialize $w\left(0\right)=0_{L_{e}}$ and $P\left(0\right)=\delta^{-1}I_{L_{e}}$4:**for** n=1,2,… **do**5:    Compute the a priori error$$e\left(n|n-1\right)=d\left(n\right)-w^{H}\left(n-1\right)u\left(n\right)$$6:    Compute the RLS gain vector$$k\left(n\right)=\frac{P(n-1)u(n)}{\lambda +u^{H}\left(n\right)P\left(n-1\right)u\left(n\right)}$$7:    Update the inverse correlation matrix using (12)8:    Form the proportionate matrix$$G\left(n-1\right)=\mathrm{diag}\{g_{1}\left(n-1\right),\ldots ,g_{L_{e}}\left(n-1\right)\}$$9:    Compute the DR contraction factor$$\alpha_{p}\left(n\right)=1-u^{H}\left(n\right)G\left(n-1\right)k\left(n\right)$$10:    Compute $\beta_{p}\left(n\right)$ using (29). If $|1-\alpha_{p}\left(n\right)|<\epsilon_{\beta }$, set $\beta_{p}\left(n\right)=N_{r}$11:    Update the coefficient vector$$w\left(n\right)=w\left(n-1\right)+\beta_{p}\left(n\right)G\left(n-1\right)k\left(n\right)e^{*}\left(n|n-1\right)-N_{r}\xi \;P\left(n\right)\;{\mathrm{sgn}}_{\epsilon }\;\left(w(n-1)\right)$$12:**end for**

In particular, the a priori error characterizes the mismatch between the desired signal and the current output, the RLS recursion updates the gain efficiently, the proportionate matrix assigns more emphasis to dominant coefficients, and the DR factor improves the utilization of the current observation. In addition, the $\ell_{1}$ regularization term helps suppress insignificant taps and thus promotes sparse UWA channel equalization.

### 3.4. Computational Complexity Analysis

The dominant per-iteration complexity of the proposed DR-$\ell_{1}$-PRLS algorithm is governed by the same matrix–vector and matrix–matrix operations as in conventional $\ell_{1}$-PRLS, namely, the gain computation in (11) and the information matrix update in (12). These operations scale as $O(L_{e}^{2})$.

Compared with $\ell_{1}$-PRLS, the DR extension in the practical form (35) only introduces the following:

- One additional scalar product $u^{H}\left(n\right)G\left(n-1\right)k\left(n\right)$ for $\alpha_{p}\left(n\right)$;
- One scalar geometric series computation for $\beta_{p}\left(n\right)$;
- A few scalar operations for the numerical safeguard.

Therefore, the dominant complexity order remains unchanged:

(40) $$C_{\mathrm{DR}\text{-}\ell_{1}\text{-}\mathrm{PRLS}}=O\left(L_{e}^{2}\right).$$

In addition to arithmetic complexity, practical deployment also depends on memory demand and hardware resources. The main memory cost comes from storing the inverse correlation matrix and several state vectors and also scales with $L_{e}^{2}$. Therefore, the proposed method is feasible for real-time implementation in moderate-size systems, but its storage requirement may become more demanding when the equalizer length is large or when hardware resources are limited.

From an implementation viewpoint, the proposed method preserves the same dominant complexity order as $\ell_{1}$-PRLS, and the extra cost introduced by data reuse is mainly a small scalar overhead. This is a favorable property for hardware implementation, since the main computation still follows the standard recursive structure of RLS-type algorithms. However, the data reuse operation increases the number of effective weight updates within each iteration. As a result, the actual processing time may increase when $N_{r}$ becomes large, even though the asymptotic complexity order is unchanged.

For real underwater acoustic systems with latency constraints, the applicability of the proposed algorithm depends on the equalizer length, the selected reuse factor $N_{r}$, the sampling rate, and the available processing hardware. In systems with moderate dimensions and a moderate $N_{r}$, real-time implementation is feasible. In contrast, for systems with very strict delay requirements, an excessively large $N_{r}$ may increase the per-iteration latency and reduce real-time suitability. Therefore, in practical deployment, $N_{r}$ should be selected by jointly considering estimation performance, memory cost, and latency requirements.

Overall, the proposed method improves steady-state estimation accuracy through data reuse while preserving the same dominant complexity order as $\ell_{1}$-PRLS. In implementation-oriented comparisons, it is therefore reasonable to place DR-$\ell_{1}$-PRLS and $\ell_{1}$-PRLS in the same $O(L_{e}^{2})$ complexity class while noting that DR-$\ell_{1}$-PRLS involves additional memory access and a moderate increase in processing time when $N_{r}$ is increased.

## 4. Simulations and Discussion

In this section, simulation experiments are conducted to evaluate the performance of the proposed DR-$\ell_{1}$-PRLS algorithm based on a channel model similar to that in [30]. A finite-state Markov–Gaussian (MG) model is adopted to characterize the block-sparse multipath structure of practical underwater acoustic (UWA) communication channels.

By generating clustered non-zero channel coefficients separated by long zero or near-zero regions, the MG model provides a reasonable statistical approximation of the multipath propagation behavior in realistic UWA environments. In particular, it captures the sparse arrival pattern and clustered multipath structure often observed in measured UWA channels. However, this model is still an idealized approximation; it does not fully describe all propagation details of real UWA channels, such as possible correlation in path amplitudes, more complicated delay-dependent fading behavior, or environmental nonstationarity.

The MG model describes the transitions between inactive (zero) taps and active (non-zero) taps through state transition probabilities. By adjusting these parameters, channels with different sparsity levels and structures can be generated.

The parameter set of the MG model is denoted by $M(L,\sigma_{m}^{2},p,q)$, where *L* is the channel length, *p* is the transition probability from an active tap to an inactive tap, and *q* is the transition probability from an inactive tap to an active tap, with p,q∈[0,1]. To generate sparse channels, the condition p>q is imposed.

The variance $\sigma_{m}^{2}$ characterizes the amplitude distribution of the active taps. Accordingly, the channel sparsity level is defined as

(41) $$S=\frac{1-p}{2-p-q}.$$

To illustrate the relationship between the sparsity measure in (41) and the resulting channel structure, discrete-time channel impulse responses (CIRs) are generated based on the Markov–Gaussian model. Their magnitude profiles are also plotted to show that the adopted model can capture the typical sparse multipath characteristics of UWA channels.

Specifically, for a given channel length *L* and parameter set $M(L,\sigma_{m}^{2},p,q)$, a two-state Markov chain is first used to generate an active/inactive state sequence along the delay axis. The corresponding tap coefficient is then drawn from a Gaussian distribution $N(0,\sigma_{m}^{2})$ if the state is active and is set to zero otherwise.

By tuning (p,q) to obtain a target sparsity level *S*, CIR vectors $h={\left[h_{0},\ldots ,h_{L-1}\right]}^{T}$ with different sparsity degrees and clustered multipath structures can be generated. In the subsequent simulations, the channel length is fixed to *L*, while different sparsity levels are considered by varying *p* and *q*.

**Figure 2:**
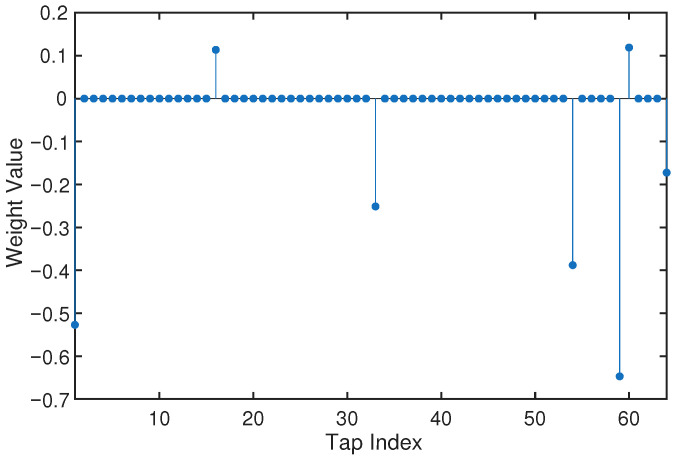
A representative channel impulse response (CIR) realization with a clustered sparse multipath structure generated by the Markov–Gaussian channel model.

This yields multiple CIR realizations for comparing different adaptive equalizers in terms of convergence accuracy and steady-state error. A representative example is shown in Figure 2, where a few dominant paths and several weaker paths appear in clusters along the delay axis.

Such a structure is consistent with the sparse multipath property of practical UWA channels, which is mainly caused by surface/bottom reflections and geometric propagation effects.

For a fair and reproducible comparison, all benchmark algorithms and the proposed method are evaluated under the same simulation setup, including with identical channel settings, equalizer length, training sequence length, and number of Monte Carlo trials. Specifically, the equalizer length is set to 64, the training sequence length is fixed at 1500, and the number of Monte Carlo runs is 200 for all algorithms.

To ensure fairness in parameter tuning, the hyperparameters of all compared methods, including the step size, forgetting factor, initialization factor, and regularization-related parameters, are optimized separately through repeated simulations under the same UWA channel conditions. The final values are selected to ensure stable convergence and representative performance for each algorithm.

Furthermore, the effects of key parameter choices on algorithm performance are examined in the subsequent simulation section through dedicated experiments. The final parameter settings of the six benchmark algorithms and the proposed method are summarized in Table 1 for reproducibility.

### 4.1. Algorithm Complexity

In this section, under the simulation setting with a transmission sequence length of N=1500 and a channel length of $L_{h}=64$, the runtime of different adaptive equalization algorithms is measured and compared. Here, the runtime is used as an engineering indicator of computational complexity.

**Figure 3:**
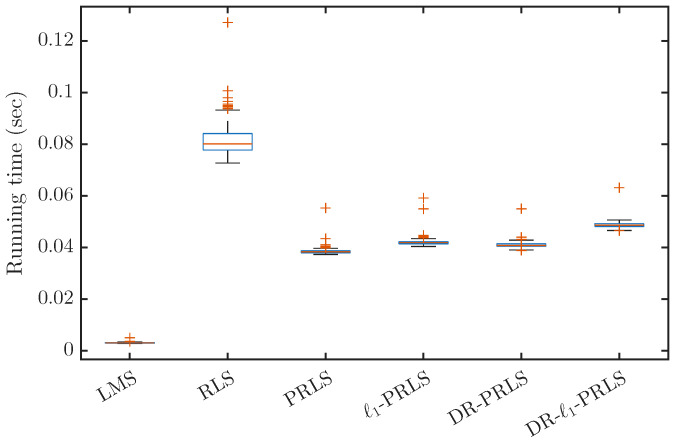
Runtime comparison of the considered adaptive equalization algorithms under the simulation setting N=1500, $L_{h}=64$.

As shown in Figure 3, LMS has the lowest average runtime, i.e., 0.00303, which is much smaller than that of the RLS-based algorithms. For the RLS-based methods, the average runtimes of RLS, PRLS, $\ell_{1}$-PRLS, DR-PRLS, and DR-$\ell_{1}$-PRLS are 0.08161, 0.03847, 0.04202, 0.04092, and 0.04868, respectively.

Compared with $\ell_{1}$-PRLS and DR-PRLS, the proposed DR-$\ell_{1}$-PRLS increases the average runtime by about 15.9% and 19.0%, respectively. Nevertheless, its runtime is still about 40.4% lower than that of the conventional RLS algorithm, indicating that the additional cost introduced by data reuse and $\ell_{1}$ regularization remains acceptable.

### 4.2. Convergence Performance

We adopt the mean squared deviation (MSD) as the performance metric to quantify the normalized mismatch between the estimated coefficients and the true ones. To be consistent with the dB scale used on the vertical axis, the MSD is defined in dB as

(42) $$\mathrm{MSD}(\mathrm{dB})=20\log_{10}\left(\frac{∥w\left(n\right)-w_{o}{∥}_{2}^{2}}{∥w_{o}{∥}_{2}^{2}}\right),$$

where w(n) denotes the estimated coefficient vector produced by the algorithm, $w_{o}$ is the reference (true) coefficient vector, and ${∥\cdot ∥}_{2}$ denotes the $\ell_{2}$ norm.

**Figure 4:**
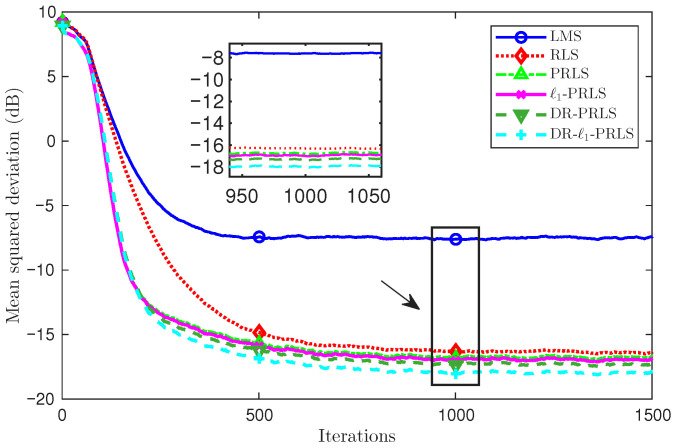
Comparison of MSD(dB) learning curves of six algorithms under the 5 dB noise condition.

**Figure 5:**
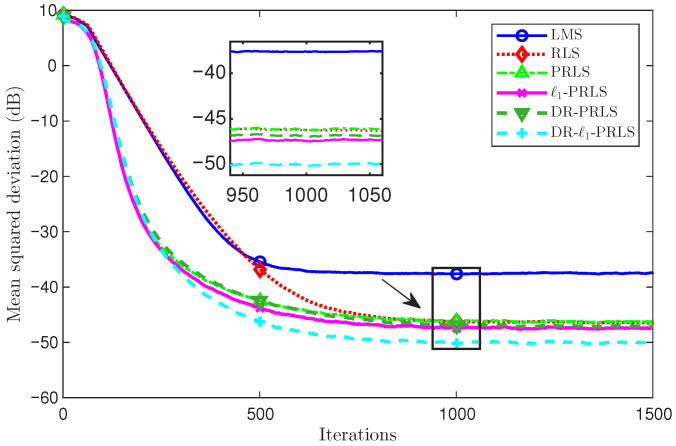
Comparison of MSD(dB) learning curves of six algorithms under the 20 dB noise condition.

Figure 4 and Figure 5 compare the MSD(dB) learning curves of the six algorithms under two noise conditions: Figure 4 corresponds to the 5 dB case and Figure 5 corresponds to the 20 dB case. In both cases, all algorithms exhibit a rapid MSD(dB) decrease in the initial stage and then gradually approach a steady state.

In Figure 5, it can be seen that all methods benefit from the reduced noise level and achieve lower MSD(dB) values than in the 5 dB case. The relative performance ordering remains essentially unchanged, with DR-$\ell_{1}$-PRLS providing the best accuracy, followed by $\ell_{1}$-PRLS and DR-PRLS, whereas LMS remains the worst.

At the 1000th iteration, which is close to the steady-state region, the MSD values of LMS, RLS, PRLS, $\ell_{1}$-PRLS, DR-PRLS, and DR-$\ell_{1}$-PRLS are −37.61 dB, −46.24 dB, −46.19 dB, −47.42 dB, −46.90 dB, and −50.08 dB, respectively. Compared with $\ell_{1}$-PRLS, DR-$\ell_{1}$-PRLS achieves an additional MSD reduction of about 2.66 dB, while the gains over DR-PRLS, PRLS, RLS, and LMS are about 3.18 dB, 3.89 dB, 3.85 dB, and 12.48 dB, respectively.

### 4.3. Tracking Performance

**Figure 6:**
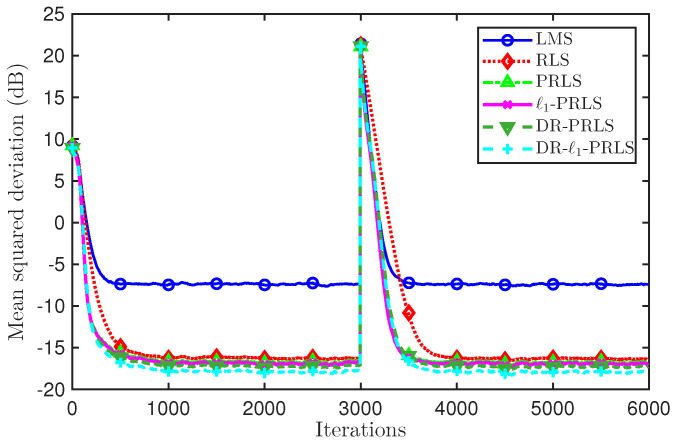
Comparison of MSD(dB) learning curves and tracking performance of six algorithms under a channel reversal ($h_{new}=-h_{old}$) at 5 dB.

**Figure 7:**
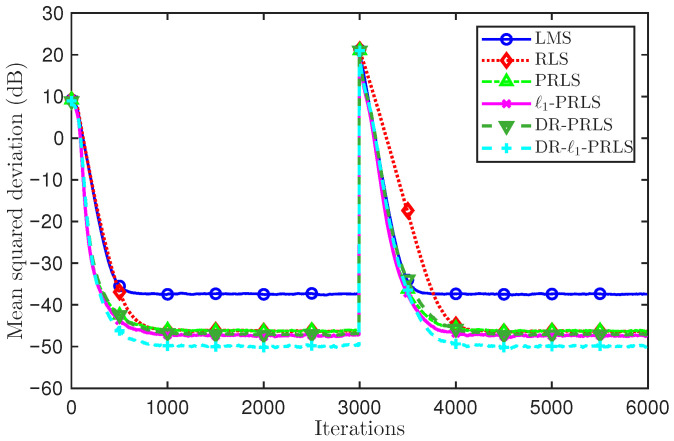
Comparison of MSD(dB) learning curves and tracking performance of six algorithms under a channel reversal ($h_{new}=-h_{old}$) at 20 dB.

To evaluate the tracking ability of the considered algorithms in a time-varying channel scenario, we further introduce an abrupt channel change in the simulation. Specifically, a channel reversal is applied during the iteration process by setting $h_{new}=-h_{old}$, which emulates a sudden change of the channel coefficients. Figure 6 and Figure 7 depict the MSD(dB) learning curves of the six algorithms (LMS, RLS, PRLS, $\ell_{1}$-PRLS, DR-PRLS, and DR-$\ell_{1}$-PRLS) under 5 dB and 20 dB, respectively, to compare their convergence and tracking behaviors.

As shown in Figure 6 (5 dB), all algorithms converge in the initial stage and exhibit a clear MSD(dB) jump when the channel reversal occurs at about iteration 3000, followed by a re-convergence process. LMS recovers more slowly and remains at a relatively high error level, whereas the RLS-based methods show faster recovery and lower MSD(dB) values. In particular, DR-$\ell_{1}$-PRLS achieves the best performance before and after the channel reversal.

At the 1000th iteration, the MSD values of LMS, RLS, PRLS, $\ell_{1}$-PRLS, DR-PRLS, and DR-$\ell_{1}$-PRLS are −7.46 dB, −16.27 dB, −16.65 dB, −16.84 dB, −17.20 dB, and −17.84 dB, respectively. Compared with $\ell_{1}$-PRLS and DR-PRLS, the proposed DR-$\ell_{1}$-PRLS provides additional MSD reductions of about 1.00 dB and 0.64 dB, respectively. After the channel reversal, at the 4000th iteration, the corresponding MSD values are −7.36 dB, −16.26 dB, −16.63 dB, −16.82 dB, −17.16 dB, and −17.79 dB, respectively, showing a similar performance ordering.

Figure 7 (20 dB) shows that all methods achieve lower MSD(dB) values under a lower-noise condition, while the transient behavior around the reversal point and the relative performance ordering remain essentially unchanged. Overall, under the abrupt time-varying channel modeled by $h_{new}=-h_{old}$, DR-$\ell_{1}$-PRLS consistently provides the best tracking performance and the lowest error level among all compared methods.

It should be noted that the channel reversal considered here is mainly used to examine the response of the algorithms to an abrupt change. In practical UWA communications, however, channel variations may also be slower and more continuous. In such cases, the proposed DR-$\ell_{1}$-PRLS is still expected to benefit from improved per-snapshot error reduction, which can help maintain a low estimation error level.

At the same time, since data reuse strengthens the dependence on the current observation, an excessively large reuse factor $N_{r}$ may reduce tracking flexibility when the channel changes continuously. Therefore, for slowly time-varying channels, $N_{r}$ and the forgetting factor λ should be jointly selected to balance estimation accuracy and tracking capability. In general, a moderately small $N_{r}$ together with a proper forgetting factor is more suitable for tracking gradual channel variations.

### 4.4. Ablation Study on DR and $\ell_{1}$ Regularization

To further clarify the source of the performance improvement achieved by the proposed DR-$\ell_{1}$-PLRS algorithm, an overall performance comparison alone is not sufficient. Although the preceding experiments have shown that the proposed method attains superior steady-state accuracy under different SNR conditions, these results do not directly reveal whether the observed gain comes from data reuse (DR), $\ell_{1}$ sparse regularization, or their combination.

Therefore, in this subsection, a dedicated 2×2 ablation study is conducted, where DR and $\ell_{1}$ regularization are treated as two binary factors. Accordingly, four variants are constructed, namely, PRLS, DR-PRLS, $\ell_{1}$-PRLS, and DR-$\ell_{1}$-PRLS, in order to systematically quantify the individual contribution of DR, the individual contribution of $\ell_{1}$ regularization, the overall joint gain when both are combined, and the interaction gain beyond simple accumulation.

**Figure 8:**
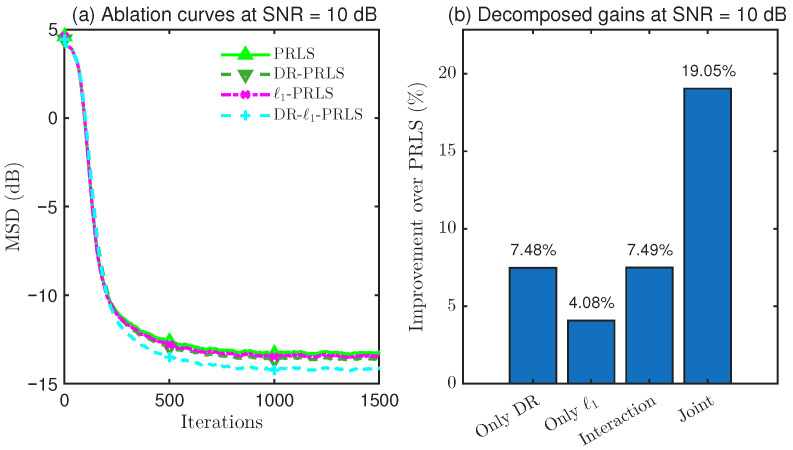
Performance comparison and gain decomposition of DR-$\ell_{1}$-PRLS and its ablated variants at SNR=10dB.

**Figure 9:**
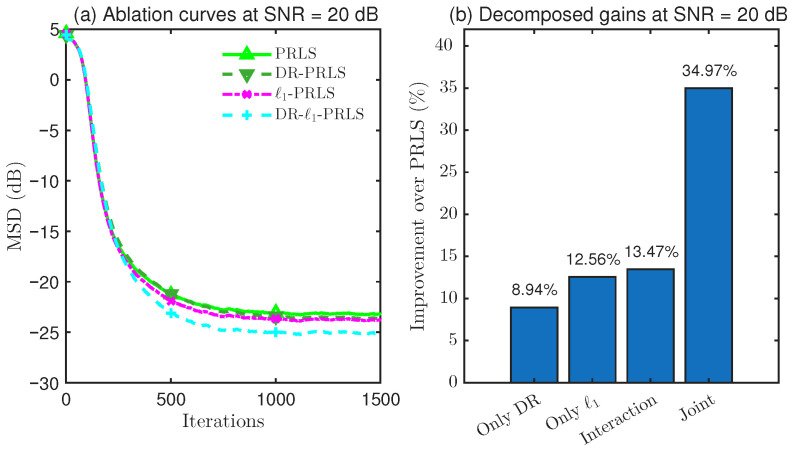
Performance comparison and gain decomposition of DR-$\ell_{1}$-PRLS and its ablated variants at SNR=20dB.

**Figure 10:**
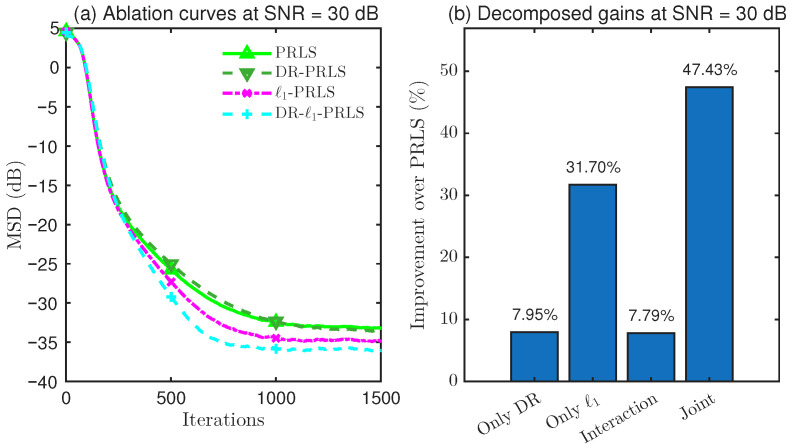
Performance comparison and gain decomposition of DR-$\ell_{1}$-PRLS and its ablated variants at SNR=30dB.

As shown in Figure 8, Figure 9 and Figure 10, the ablation results at SNR=10, 20, and 30dB consistently indicate that the DR mechanism and the $\ell_{1}$ regularization term each provide a distinct contribution to the steady-state performance improvement over PRLS. In all tested cases, the joint DR-$\ell_{1}$ design achieves the greatest overall gain, which verifies the complementarity of these two components in the proposed algorithm.

More specifically, at 10 dB, DR provides a larger standalone gain than $\ell_{1}$ regularization (7.48% vs. 4.08%), whereas, at 20 dB and 30 dB, the contribution of $\ell_{1}$ regularization becomes increasingly more pronounced (12.56% and 31.70%, respectively), suggesting that sparsity exploitation becomes more effective as the SNR increases. In addition, the joint gains achieved by combining DR and $\ell_{1}$ regularization are 19.05%, 34.97%, and 47.43% at 10, 20, and 30 dB, respectively, all of which exceed the sum of the two individual gains. This leads to positive interaction gains of 7.49%, 13.47%, and 7.79%, respectively. The two-way ANOVA further confirms that the interaction effect is statistically significant in all cases, which verifies that the superiority of DR-$\ell_{1}$-PRLS comes not only from the individual effects of DR and $\ell_{1}$ regularization, but also from their positive synergy.

The main contribution of this work is to integrate data reuse (DR) and sparse $\ell_{1}$ regularization into the PRLS framework. Theoretically, their individual roles and positive interaction are clarified through a dedicated ablation study; engineering-wise, a simple and deployable DR-$\ell_{1}$-PRLS recursion is developed to improve the overall adaptation performance and steady-state accuracy.

### 4.5. Sensitivity Analysis of Key Parameters

To improve the reproducibility of the reported results, the hyperparameters of the proposed DR-$\ell_{1}$-PRLS algorithm were selected using a simple grid-search procedure before the main comparative experiments. Specifically, the data reuse factor and the regularization weight were searched over $N_{r}\in \left\{1,2,3,5,10\right\}$ and $\xi \in \{0,10^{-4},10^{-3},10^{-2}\}$, respectively.

For each candidate setting, simulations were conducted under the same channel model and noise conditions as those used in the main experiments, and the average MSD performance was adopted as the primary selection criterion. When multiple candidate settings yielded similar MSD values, the configuration with the smaller $N_{r}$ was preferred to reduce computational complexity, while a smaller ξ was preferred only when comparable performance could be achieved, so as to avoid excessive shrinkage bias.

**Figure 11:**
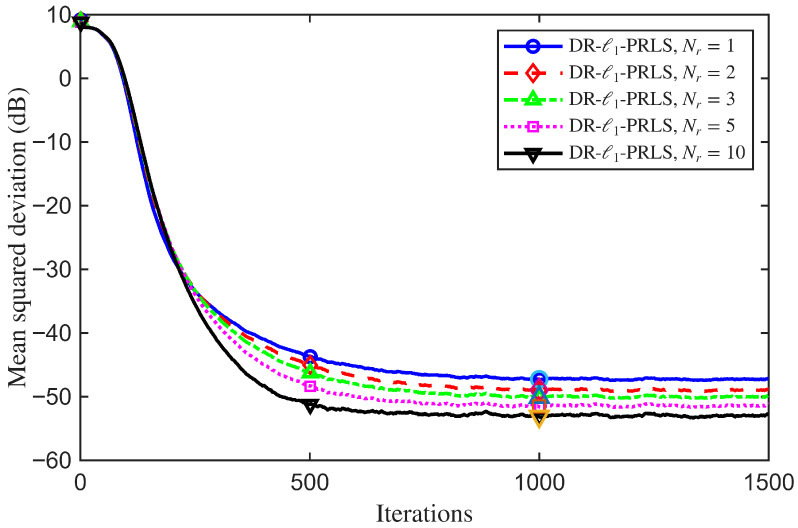
MSD learning curves of the proposed DR-$\ell_{1}$-PRLS algorithm for different data reuse factors $N_{r}$.

**Figure 12:**
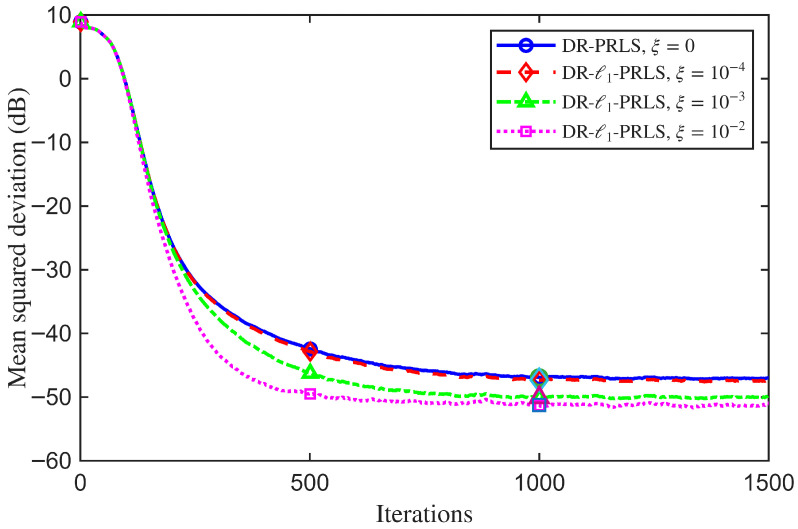
MSD learning curves of the proposed DR-$\ell_{1}$-PRLS algorithm for different regularization weights ξ.

Since $\xi =10^{-2}$ provided the best steady-state MSD among the tested values under the adopted simulation setting, $N_{r}=3$ and $\xi =10^{-2}$ were selected as the nominal parameter settings used throughout the main simulations. The sensitivity analyses reported in Figure 11 and Figure 12 further illustrate the influence of these two parameters on the convergence behavior and steady-state performance.

To further illustrate the effect of the data reuse factor on the proposed DR-$\ell_{1}$-PRLS algorithm, a sensitivity analysis with respect to $N_{r}$ is carried out. With the other system and algorithmic parameters fixed at their nominal settings, comparative simulations are performed for different values of $N_{r}$, and the corresponding MSD learning curves are used to show the influence of the reuse factor on the convergence behavior and steady-state performance of the proposed algorithm.

Figure 11 shows the MSD learning curves of the DR-$\ell_{1}$-PRLS algorithm for different values of $N_{r}$. Changing the data reuse factor clearly affects both the convergence rate and the steady-state mismatch. This shows that $N_{r}$ is an important parameter for the algorithm performance. At the 1000th iteration, the MSD values for $N_{r}=1,2,3,5,$ and 10 are −47.26 dB, −48.98 dB, −50.08 dB, −51.54 dB, and −53.10 dB, respectively.

In general, increasing $N_{r}$ helps the algorithm use the current observation more effectively and improves the estimation accuracy. When $N_{r}$ increases from 1 to 10, the MSD is reduced by about 5.83 dB. This confirms the effectiveness of the data reuse strategy. However, when $N_{r}$ becomes too large, the performance gain gradually saturates, while the computational complexity increases accordingly.

To further illustrate the effect of sparse regularization on the proposed DR-$\ell_{1}$-PRLS algorithm, a sensitivity analysis with respect to ξ is conducted. With the other system and algorithmic parameters fixed at their nominal settings, comparative simulations are performed for different values of ξ, and the corresponding MSD learning curves are used to show the influence of the regularization weight on the convergence behavior and steady-state performance of the proposed algorithm.

Figure 12 shows that the regularization weight ξ has a clear effect on both the convergence behavior and the steady-state mismatch. When ξ=0, the algorithm reduces to DR-PRLS without explicit sparse regularization, and the steady-state performance is relatively poor. At the 1000th iteration, the MSD values for ξ=0, $10^{-4}$, $10^{-3}$, and $10^{-2}$ are −46.90 dB, −47.31 dB, −50.08 dB, and −51.23 dB, respectively.

As ξ increases, the sparsity-promoting effect becomes stronger, and the estimation accuracy is improved in this experiment. Compared with ξ=0, the MSD is reduced by about 4.33 dB when $\xi =10^{-2}$. Among the tested values, $\xi =10^{-2}$ gives the best steady-state performance under the current simulation setting. These results show that a proper $\ell_{1}$ regularization term is helpful for the proposed algorithm.

The impact of the channel sparsity level on the proposed DR-$\ell_{1}$-PRLS algorithm is also studied under different channel conditions. In this experiment, the underwater acoustic channel is generated by the adopted Markov–Gaussian model, where the sparsity level is described by the indicator *S*. All other parameters are kept fixed, and simulations are carried out for several representative values of *S*.

Under a fixed $N_{r}=3$ and $\xi =10^{-2}$, the effect of different channel sparsity levels on the performance of the proposed DR-$\ell_{1}$-PRLS algorithm is further investigated.

**Figure 13:**
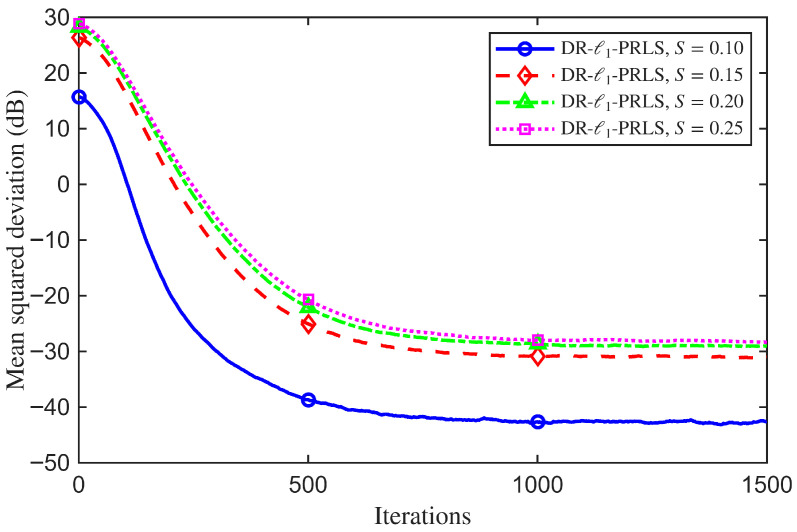
MSD learning curves of the proposed DR-$\ell_{1}$-PRLS algorithm under different channel sparsity levels.

Figure 13 shows the MSD learning curves of the proposed DR-$\ell_{1}$-PRLS algorithm under different channel sparsity levels. The channel sparsity level has a clear effect on both the convergence behavior and the steady-state mismatch. At the 1000th iteration, the MSD values for S=0.10, 0.15, 0.20, and 0.25 are −42.67 dB, −30.82 dB, −28.65 dB, and −27.97 dB, respectively.

In general, a smaller value of *S* corresponds to a sparser channel. In this case, the proposed algorithm can exploit the sparse structure more effectively and achieve higher estimation accuracy. Compared with S=0.25, the MSD is reduced by about 14.70 dB when S=0.10. This result shows that the algorithm performs much better in sparser channels.

These results further show that the benefit of the $\ell_{1}$ regularization term is more significant in sparse or cluster-sparse environments. Therefore, in practical applications, the reuse factor $N_{r}$ and the regularization weight ξ should be selected jointly according to the channel sparsity level, so as to obtain a proper tradeoff between convergence performance and computational complexity.

### 4.6. Noise Robustness and Equalization Performance

This section establishes an end-to-end digital communication link equalization simulation to evaluate the equalization capability and noise robustness of the proposed algorithm in sparse multipath underwater acoustic (UWA) channels, with a focus on its performance under different signal-to-noise-ratio conditions.

In the simulation, QPSK (4-PSK) modulation with Gray mapping is employed at the transmitter. To support adaptive equalizer training at the receiver, a pilot sequence occupying 10% of the symbol stream is inserted as the training segment. The transmitted signal is filtered by a sparse multipath channel with length $L_{h}=64$, and additive white Gaussian noise (AWGN) is then injected. The noise level is controlled by $E_{b}/N_{0}$, which is swept from 0 to 20 dB to systematically evaluate bit error performance under different SNR conditions.

At the receiver, an adaptive equalizer with 128 taps is constructed and trained using LMS, RLS, PRLS, $\ell_{1}$-PRLS, DR-PRLS, and DR-$\ell_{1}$-PRLS, respectively. The equalized output is demodulated and compared with the original transmitted bits to compute the bit error rate (BER), which is used as the main performance metric. The BER is defined as

(43) $$P_{b}=\frac{e_{b}}{e_{s}}$$

where $e_{b}$ denotes the number of erroneous bits and $e_{s}$ denotes the total number of transmitted bits.

**Figure 14:**
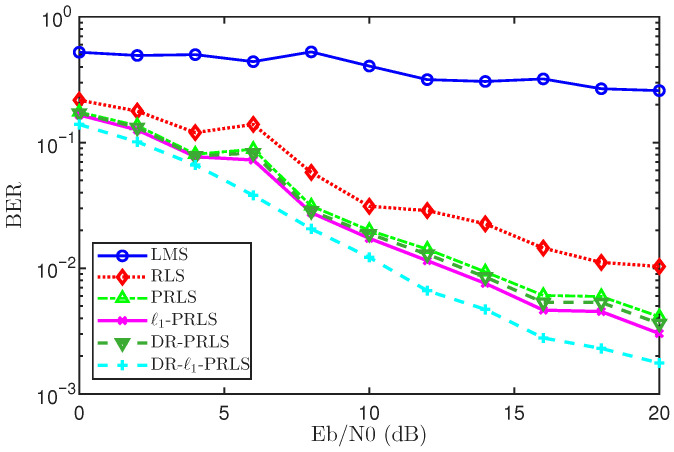
BER performance comparison versus $E_{b}/N_{0}$ for different equalization algorithms over a sparse UWA multipath channel.

By plotting the BER as a function of $E_{b}/N_{0}$, the equalization performance and noise robustness of both conventional and sparsity-aware adaptive algorithms can be directly compared over the sparse UWA multipath channel. The corresponding BER results are shown in Figure 14. Overall, the proposed DR-$\ell_{1}$-PRLS algorithm achieves the best BER performance among the tested methods.

Specifically, at $E_{b}/N_{0}=0$, 10, and 20 dB, the BERs of DR-$\ell_{1}$-PRLS are about $1.39\times 10^{-1}$, $1.22\times 10^{-2}$, and $1.76\times 10^{-3}$, respectively. Under the same conditions, the BERs of $\ell_{1}$-PRLS are about $1.66\times 10^{-1}$, $1.73\times 10^{-2}$, and $3.03\times 10^{-3}$, respectively. These results show that the proposed method can effectively reduce the BER, and the advantage becomes more evident in the medium- and high-SNR regions.

For each $E_{b}/N_{0}$ point, the BER is evaluated over 200 independent Monte Carlo trials. In each trial, the transmitted bit stream and the AWGN realization are regenerated independently. The final BER is obtained from the aggregated error counts over all trials, which ensures a fair and statistically reliable comparison.

**Figure 15:**
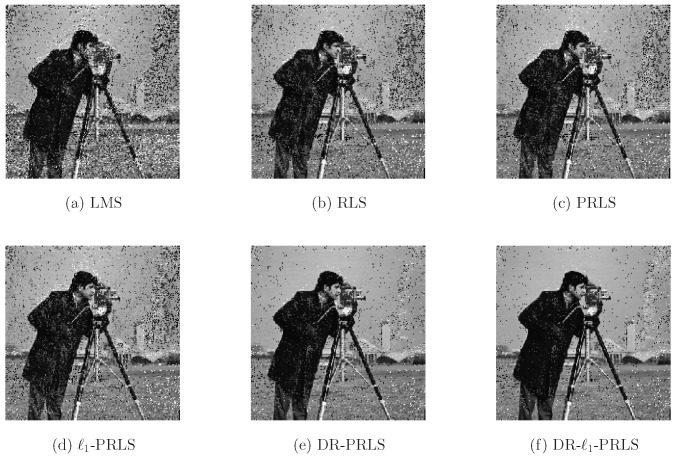
Visual comparison of image reconstruction quality under different equalization algorithms.

To provide a more intuitive demonstration of the equalization quality, the detected bitstream is further used for image reconstruction after symbol detection and bit decision. The reconstructed images obtained with different equalization methods are shown in Figure 15. These visual results complement the BER analysis from a perceptual perspective and illustrate the impact of each algorithm on practical information recovery.

In addition, the main simulation settings and frame parameters are summarized in Table 1. Overall, this section evaluates the proposed DR-$\ell_{1}$-PRLS algorithm in sparse UWA multipath channels through both BER curves and image reconstruction results, providing a comprehensive assessment of its equalization performance and noise robustness.

## 5. Conclusions and Future Work

To improve adaptive equalization over sparse channels, this paper investigated and compared LMS, RLS, PRLS, $\ell_{1}$-PRLS, DR-PRLS, and the proposed DR-$\ell_{1}$-PRLS. The simulation results show that the proposed method achieves the best overall performance in terms of both BER and MSD.

Specifically, DR-$\ell_{1}$-PRLS achieves the lowest BER at $E_{b}/N_{0}=0$, 10, and 20 dB, with values of $1.392574\times 10^{-1}$, $1.220703\times 10^{-2}$, and $1.764297\times 10^{-3}$, respectively. It also attains the lowest MSD at the 1000th iteration, i.e., −50.0815 dB, which is lower than that of $\ell_{1}$-PRLS and DR-PRLS.

These results confirm that the combination of data reuse and $\ell_{1}$ regularization is effective for sparse channel equalization, and that the proposed DR-$\ell_{1}$-PRLS algorithm provides superior equalization accuracy and estimation performance.

Future work will address several limitations of the proposed method: First, the algorithm is sensitive to parameter selection, especially the data reuse factor $N_{r}$, the forgetting factor λ, and the regularization weight ξ. Although proper settings can improve the performance, parameter tuning is still needed under different channel sparsity levels and noise conditions. Future work will therefore study adaptive $N_{r}$ selection and more general self-tuning parameter strategies.

Second, the performance of the proposed algorithm under very-low-SNR conditions still needs further study. In such cases, severe noise may reduce the reliability of the estimation error and weaken the effect of sparse regularization. This may degrade both the convergence behavior and the steady-state performance. Future work will consider more systematic evaluation and more robust update schemes for low-SNR scenarios.

Third, the proposed method is mainly designed for sparse or cluster-sparse channel estimation. Its advantage may be less obvious for non-sparse channels. When the channel does not exhibit a clear sparse structure, the $\ell_{1}$ regularization term may offer limited benefit and may even introduce extra bias. Future work will therefore consider online sparsity estimation, sparsity-aware switching schemes, and hybrid regularization methods.

In addition, practical deployment still requires further study. The main implementation cost comes from recursive matrix updates, repeated weight updates caused by data reuse, and the storage of internal state variables such as the inverse correlation matrix. Therefore, both memory overhead and hardware resources should be considered in real-time implementation.

For systems with strict delay constraints, a large $N_{r}$ may increase the per-iteration processing time, although it can improve the estimation performance. Therefore, in practical deployment, $N_{r}$ should be selected by jointly considering performance and latency requirements.

Future research will consider fast recursions, partial updates, and low-rank approximations to reduce the computational cost and memory demand. The proposed method will also be extended to more practical scenarios, including MIMO-UWA systems, and further tested under colored noise, impulsive interference, and model mismatch, with additional verification on hardware-oriented platforms.

## Data Availability

The original contributions presented in this study are included in the article. Further inquiries can be directed to the corresponding author.
